# HEXACO traits, emotional intelligence, and mental health: evidence from Chinese music education and implications for personality–stress interactions

**DOI:** 10.3389/fpsyg.2025.1421484

**Published:** 2025-04-29

**Authors:** Jing Jing

**Affiliations:** School of Music and Dance, Fuyang Normal University, Fuyang, Anhui, China

**Keywords:** HEXACO personality traits, emotional intelligence, stress management, mental health, music education

## Abstract

**Introduction:**

This study examines the intricate relationship between HEXACO personality dimensions, specifically honesty-humility (H-H), emotional intelligence, stress, and mental health, within the context of music education in China.

**Methods:**

Utilizing the PROCESS Procedure for SPSS, developed by Andrew F. Hayes, we analyzed data from a sample of 746 music students aged 18–35 years, enrolled in undergraduate to graduate-level programs. The mediational model (Model 15) identified trait emotional intelligence as the mediator (M), with stress as the independent variable (X) and mental health as the dependent variable (Y), while considering H-H as a moderating variable (W).

**Results:**

Our findings demonstrate that trait emotional intelligence significantly mediates the effect of stress on mental health, with the H-H dimension moderating this mediation, thereby enhancing the protective effect of emotional intelligence against stress-induced negative mental health outcomes.

**Discussion:**

These results suggest practical applications for music education, indicating that integrating emotional intelligence training into curricula could be beneficial for improving stress management and mental health among students. Furthermore, recognizing H-H as a moderating factor highlights the importance of developing tailored support strategies to address the unique pressures faced by music students, thereby fostering a more supportive educational environment that is conducive to both academic achievement and personal growth.

## Introduction

In recent years, the relationship between personality traits, emotional intelligence, stress, and mental health has gained significant attention within psychological and educational research (Wang et al., 2024; Zhang and Hu, 2024). However, within the specialized domain of music education, where emotional and creative demands are uniquely intertwined with the educational process, these relationships remain underexplored (Chen, 2024). Despite the recognized importance of emotional intelligence in managing stress and promoting mental wellbeing, there is a lack of research specifically addressing how personality traits, particularly those in the HEXACO model, interact with these factors in the context of music education.

This study addresses this gap by focusing on the HEXACO personality dimension of honesty-humility (H-H) and its interaction with stress and mental health outcomes, mediated by trait emotional intelligence, among music students in China. Music education, inherently rich in emotional expression, offers a distinct environment for exploring how individual differences in personality traits affect students’ ability to manage stress and maintain mental health (Nwokenna et al., 2022).

While the HEXACO model has been extensively studied about various psychological outcomes, its application within the context of music education and its interaction with emotional intelligence have been limited (Li et al., 2023). Honesty-humility, a dimension encompassing sincerity, fairness, avoidance of greed, and modesty, may significantly impact how students experience stress and utilize emotional intelligence to protect their mental health in the demanding environment of music education (Pilch, 2023).

Moreover, exploring these relationships in a specific educational context, such as music training, may yield insights that are also relevant to broader psychological theory (Chen, 2023). The interplay between dispositional traits, such as H-H, and emotional functioning under stress may reflect more general mechanisms of adaptation and self-regulation. While the present study is situated within the domain of music education, the theoretical framework it engages may contribute to understanding personality–stress–health dynamics in other high-demand contexts, as Quality of Life (López-González and Topa, 2024), organizational justice (Travis et al., 2024) or working life (Mellor and Elliott, 2025).

In this sense, the current research aims not only to inform practice and support the wellbeing of music students but also to contribute to the broader literature on personality and mental health. Investigating these constructs in an emotionally intense educational environment may help reveal patterns and interactions that extend beyond the immediate sample, offering directions for future research across educational and occupational settings.

This study proposes that H-H moderates the relationship between stress and mental health, mediated by emotional intelligence, offering a novel perspective on how personality traits can influence educational and psychological outcomes. By addressing this research gap, the study aims to provide practical implications for educators and policymakers in designing targeted interventions that enhance mental wellbeing and academic success in music education.

Through this investigation, which focuses on Chinese music students in tertiary education, the research aims to contribute to the broader discourse on stress, mental health, and emotional intelligence in the educational context. The findings are expected to inform strategies that promote a supportive educational environment, ultimately aiding in the holistic development of students in the field of music education.

## Literature review

### Mental health and Chinese music students’ stress

The psychological wellbeing of music students in China has become an increasingly critical area of concern as educational paradigms shift (Sun, 2022). While stress is inherent in rigorous academic environments, the intense demands of music education—characterized by fierce competition, extensive practice routines, and the pressures of public performances—significantly elevate stress levels among these students. Although some level of stress can motivate students to strive for excellence, the predominantly high levels of stress observed among Chinese music students often pose serious threats to their mental health (Jääskeläinen, 2023).

However, the existing literature often focuses on the prevalence of mental health issues without thoroughly exploring the underlying causes or the complex interplay between stress and mental health in this unique context. Cultural factors further complicate this issue. In China, the cultural valorization of academic and musical success exacerbates these pressures, prompting students to pursue an unrelenting quest for perfection (Kai, 2012). This cultural backdrop, which prioritizes academic achievement over individual wellbeing, often leads to the neglect of mental health issues, either through stigma or lack of awareness (Qi, 2023).

While several studies have highlighted the detrimental impact of these stressors on mental health, revealing issues such as anxiety, depression, and other forms of psychological distress among Chinese music students (Demirbatir, 2012; Kamza and Bazarbekova, 2023; Wristen, 2013), there remains a gap in understanding the mechanisms through which stress impacts mental health. Specifically, the role of personality traits and emotional intelligence in moderating or mediating these effects has not been sufficiently explored. Addressing this gap is crucial for developing targeted interventions that can mitigate these stressors and improve mental health outcomes among music students in China.

The present study aims to address this gap by investigating the impact of stress on mental health among Chinese music students, with a focus on the potential mediating role of Trait Emotional Intelligence (TEI) and the moderating role of the H-H dimension of the HEXACO personality model. By exploring these factors, this study aims to provide a more comprehensive understanding of the factors that contribute to mental health outcomes in this population, offering insights into potential strategies for enhancing student wellbeing.

### Trait emotional intelligence as a mediator between stress and mental health

Trait Emotional Intelligence (TEI) has been increasingly recognized as a critical factor in mediating the relationship between stress and mental health. TEI encompasses the ability to perceive, use, understand, and manage emotions, which can act as a buffer against the negative effects of stress. Studies have shown that students with higher levels of TEI are better equipped to manage stress and are less likely to experience negative mental health outcomes, such as anxiety and depression (Fteiha and Awwad, 2020; Zarei et al., 2019).

However, the literature presents mixed findings on the role of TEI across different educational contexts. For example, while some studies have found that higher TEI levels are associated with better adaptation and wellbeing among students, others have indicated that TEI does not significantly predict competence or academic performance, suggesting that other factors, such as adversity quotient, might play a more crucial role (Widodo et al., 2022). This discrepancy underscores the need for further research to elucidate the conditions under which TEI functions as an effective mediator in the stress-mental health relationship.

In the context of university students, several studies have confirmed the mediating role of TEI between stress and mental health, suggesting that interventions aimed at enhancing TEI could be beneficial in reducing stress and improving mental health outcomes (Ewaiwi et al., 2024). These findings underscore the importance of integrating TEI training into educational curricula to better equip students with the emotional skills necessary to manage stress and maintain mental wellbeing. However, the specific dynamics of how TEI interacts with personality traits, such as H-H, in this mediation process remain underexplored, particularly in the context of music education.

### Honesty-humility as a moderator in the stress-mental health relationship

Personality traits, particularly those within the HEXACO model, are often considered foundational factors that influence how individuals cope with stress. The H-H dimension, in particular, is characterized by traits such as sincerity, fairness, greed avoidance, and modesty, which may play a crucial role in moderating the impact of stress on mental health. Individuals high in H-H are generally sincerer, fair, and modest, while those low in this trait are more likely to exhibit manipulativeness, deceit, and pretentiousness.

While direct evidence on the moderating role of H-H in the stress-mental health relationship is limited, related research suggests its potential significance. For instance, studies have shown that personality traits promoting positive self-regard and kindness, such as those encompassed by H-H, can buffer the negative impacts of stress (Zhang et al., 2016). Furthermore, the emphasis on fairness and sincerity within the H-H dimension could influence how individuals perceive and respond to stress, potentially leading to more adaptive coping strategies and better mental health outcomes.

The literature on emotional intelligence and coping styles further supports the potential moderating role of H-H. For example, Sun and Lyu (2022) highlighted the importance of emotional and interpersonal skills in managing stress, suggesting that individuals high in H-H may be more effective in leveraging these skills. Although there is a need for direct empirical studies to explore H-H’s moderating role in the stress-mental health relationship, the existing literature on personality traits and stress management provides a strong theoretical foundation for this line of inquiry.

### Honesty-humility and trait emotional intelligence: a moderated mediation model

Building on the previous sections, the current study proposes that H-H not only moderates the direct relationship between stress and mental health but also moderates the indirect relationship mediated by TEI. This moderated mediation model suggests that individuals with high levels of H-H may utilize their TEI more effectively in managing stress, leading to better mental health outcomes.

Empirical studies have demonstrated that TEI can influence coping styles and mental health outcomes, with higher TEI levels being linked to a preference for problem-oriented coping strategies and better mental health (Molero-Jurado et al., 2021). However, the role of H-H in this process remains underexplored. It is hypothesized that H-H, by promoting sincerity, fairness, and modesty, could enhance the effectiveness of TEI in stress management, thus further protecting against negative mental health outcomes.

For instance, in educational settings, students with high TEI who also score high on H-H might be better equipped to manage stress through empathetic understanding and emotional regulation, fostering resilience and positive mental health. This hypothesis aligns with findings that higher TEI levels correlate with lower stress levels, particularly in demanding academic environments such as medical schools (Fiorilli et al., 2020).

The present study aims to empirically test this moderated mediation model, offering new insights into the intricate relationship between personality traits, emotional intelligence, stress, and mental health. While the research is grounded in the context of Chinese music education, where students often face high emotional demands and performance pressure, the proposed model engages theoretical constructs that may hold relevance beyond this specific population. By investigating how H-H moderates the stress–mental health relationship through emotional intelligence, this study aims not only to inform targeted interventions in music education but also to contribute to a broader understanding of personality–emotion dynamics in psychologically demanding environments.

## Hypotheses

*H1*: Students’ stress will predict students’ mental health.

*H2*: Trait Emotional intelligence will mediate between Students’ Stress and Mental Health.

*H3*: H-H will moderate the direct relationship between Students’ Stress and Mental Health.

**Figure 1:**
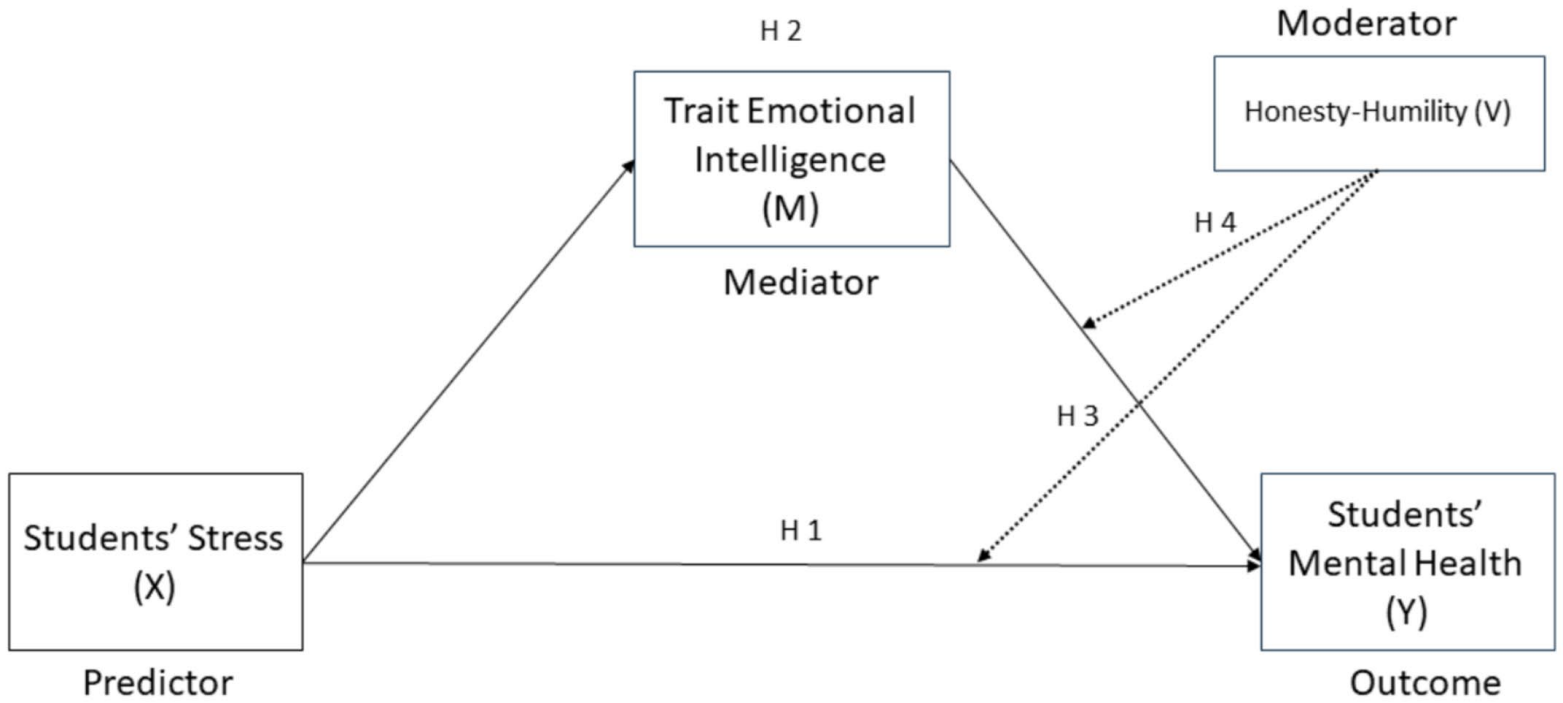
Theoretical model of the hypothesized moderated mediation. Arrows indicate the proposed paths tested in the study, where stress (X) influences mental health (Y) both directly and indirectly through trait emotional intelligence (M). Honesty-Humility (V) is hypothesized to moderate both the direct and indirect effects. Dashed lines represent moderation paths (H3 and H4).

*H4*: H-H will moderate the indirect relationship between Students’ Stress and Mental Health mediated by Emotional intelligence. The complete model of hypotheses is displayed in Figure 1.

## Method

### Participants

This investigation focused on a group of Chinese Music students, totaling 746 individuals, ranging in age from 18 to 35 years (average age = 26.98 years; standard deviation = 4.76), of whom 59.9% were female. These students were enrolled in Tertiary Education, both at undergraduate and graduate levels, in conservatories, Universities, and Colleges with a department or School of music. The majority were pursuing a Bachelor of Music (50.1%), while others were pursuing a Master of Music (25.2%). Additionally, some were enrolled in doctoral programs, including the Doctor of Musical Arts (DMA) and the Ph.D. in Music (24.4%). The fields of specialization were performance, music education, composition, musicology, music technology, and conducting. Regarding their professional activities, the majority of the participants (79.1%) were only studying, while 19.4% developed a part-time professional activity, and the rest were searching for a professional activity. The participants for this study were selected through a convenience sampling method, which involved recruiting students from various conservatories, universities, and colleges with music departments in China. While this approach enabled the efficient collection of data from a specific population, it may introduce certain biases, particularly related to the sample’s representativeness. For instance, students who are more engaged or have a stronger connection to their institutions might have been more likely to participate, potentially leading to an overrepresentation of highly motivated individuals. Additionally, the sample’s gender distribution, with a higher percentage of female participants, might influence the generalizability of the findings. The diversity in fields of specialization and educational levels adds depth to the sample, but it also introduces variability that could affect the consistency of the results. Future research should consider employing random sampling methods and including a broader demographic range to mitigate these biases and enhance the robustness of the findings.

### Procedure

The recruitment of participants was achieved through postings on bulletin boards and the universities’ and conservatories’ online networks. As a Music student, the author actively disseminated the information through social networks. These postings succinctly outlined the primary objective of the research and highlighted that involvement was entirely voluntary, with the option for participants to opt out at any time without repercussions, and clarified that there would be no monetary reward for participation. Contact information for the lead researcher was made available through a WeChat number for potential participants to make further inquiries. The Institutional Review Board of Fu Yang Normal University approved the design before its implementation. Those who reached out via this contact method were provided with comprehensive instructions on how to complete the survey. The survey began with three questions to confirm informed consent, and only those who affirmed their consent were permitted to proceed to the subsequent portion, which included completing various survey scales. Originally, the sample size at the outset was 789 individuals; however, 43 individuals were excluded from the analysis because they did not complete the survey in its entirety.

### Instruments

#### Students’ stress

The Perceived Stress Scale (PSS) is a self-reported 10-item scale. The 10-item version (PSS-10) has demonstrated good reliability and validity, with Cronbach’s *α* ranging from 0.78 to 0.91 and test–retest reliability coefficients of 0.55 to 0.80 (Cohen et al., 1983). The SCPSS-10, the Chinese version of the PSS-10 (Lu et al., 2017), has shown good reliability and validity with a Cronbach’s α of 0.86 (Leung et al., 2010). Participants rated how often they experienced each item over the past month on a 5-point Likert scale, ranging from 1 (never) to 5 (very often). The higher scores indicate greater levels of perceived stress. Examples of items are: “In the last month, how often have you been upset because of something that happened unexpectedly?” and “In the last month, how often have you been unable to control the important things in your life?”

#### Trait emotional intelligence

The Short-Form Trait Emotional Intelligence Questionnaire (TEIQue-SF) consists of 30 items, serving as a condensed version of the comprehensive Trait Emotional Intelligence Questionnaire (Petrides and Furnham, 2000). This instrument is designed to evaluate individuals’ self-assessed emotional abilities and their capacity to manage and perceive emotions adaptively. Illustrative items from the questionnaire are “I’m usually able to influence the way other people feel” and “I often pause and think about my feelings,” which participants respond to using a seven-point Likert scale. The aggregation of these item responses yields an overall score representing the respondent’s trait of emotional intelligence (Cooper and Petrides, 2010).

#### Honesty-humility

The HEXACO-60, introduced by Ashton and Lee (2009), represents a concise iteration of the HEXACO personality inventory, comprising 60 items. This abridged version encompasses six domains—honesty-humility, emotionality, extraversion, agreeableness, conscientiousness, and openness to experience—with each domain featuring ten items subdivided into four distinct facets. Responses to these items are measured using a five-point Likert scale. The H-H dimension includes subdimensions. The Sincerity scale evaluates individuals’ inclination towards authenticity in social interactions, distinguishing those who may use flattery for personal gain from those who prefer honesty and are resistant to manipulating others.

The Fairness scale measures one’s propensity to eschew dishonesty and unethical behavior. Individuals scoring low may resort to deceit or theft for personal benefit, while those scoring high demonstrate a commitment to ethical principles, refraining from exploiting others or society. The Greed Avoidance Scale gauges the level of disinterest in acquiring opulent wealth or symbols of high status. It contrasts those who seek and flaunt affluence with those who are indifferent to wealth and societal status as primary motivators. Lastly, the Modesty scale determines the degree to which individuals are humble and unpretentious. Those with low scores may perceive themselves as superior and deserving of special rights, whereas high scorers regard themselves as equal to others, without entitlement to privileged treatment. The stability of the structure and its psychometric properties have been tested for 18 countries, including China, with adequate findings (García et al., 2022). The Chinese simplified version of the scale is available at https://hexaco.org/hexaco-inventory. Examples of items are: “If I want something from someone, I will laugh at their jokes, even if they are not funny,” and “I would not try to please someone just to get their favors.”

#### Mental health

The Positive Mental Health Scale (PMH-scale), as introduced by Lukat et al. (2016), is a nine-item instrument designed to measure the emotional dimensions of wellbeing, albeit without direct reference to established theories of wellbeing. This scale was designed to capture a unified dimension of positive emotional states indicative of good mental health. Individuals participating in the assessment are asked to evaluate statements such as “I am in good physical and emotional condition” or “I am often carefree and in good spirits” using a four-point scale, where 0 signifies strong disagreement and 3 signifies strong agreement. The scale is noted for its robust psychometric characteristics. In studies involving various demographics, including students and distinct patient cohorts, the PMH scale consistently demonstrated a single-factor structure, emphasizing its efficacy in gauging positive mental health (Bieda et al., 2017). The Chinese adaptation of the scale was used (Wu et al., 2020).

The selection of these instruments was guided by their established reliability, validity, and relevance to the constructs being measured in this study. The Perceived Stress Scale (PSS-10) was selected due to its widespread use and robust psychometric properties, particularly its adaptability to diverse cultural contexts, as demonstrated by its validated Chinese version. This ensures that the measurement of stress levels among Chinese music students is both reliable and culturally appropriate. The Short-Form Trait Emotional Intelligence Questionnaire (TEIQue-SF) was selected for its efficiency in capturing key aspects of emotional intelligence, which is central to understanding the mediating role of emotional intelligence in the relationship between stress and mental health (Al-Dassean, 2023). The HEXACO-60 was chosen to measure H-H due to its comprehensive and empirically validated framework for assessing personality traits, including its tested reliability across various cultures (Abbasi et al., 2025), including China. Finally, the Positive Mental Health Scale (PMH-scale) was selected for its focus on the positive dimensions of mental health, which aligns with the study’s aim to assess not just the absence of distress but the presence of wellbeing. These instruments collectively provide a thorough and culturally sensitive assessment of the key variables under investigation, ensuring that the study’s findings are both robust and meaningful.

### Data analyses

**Figure 2:**
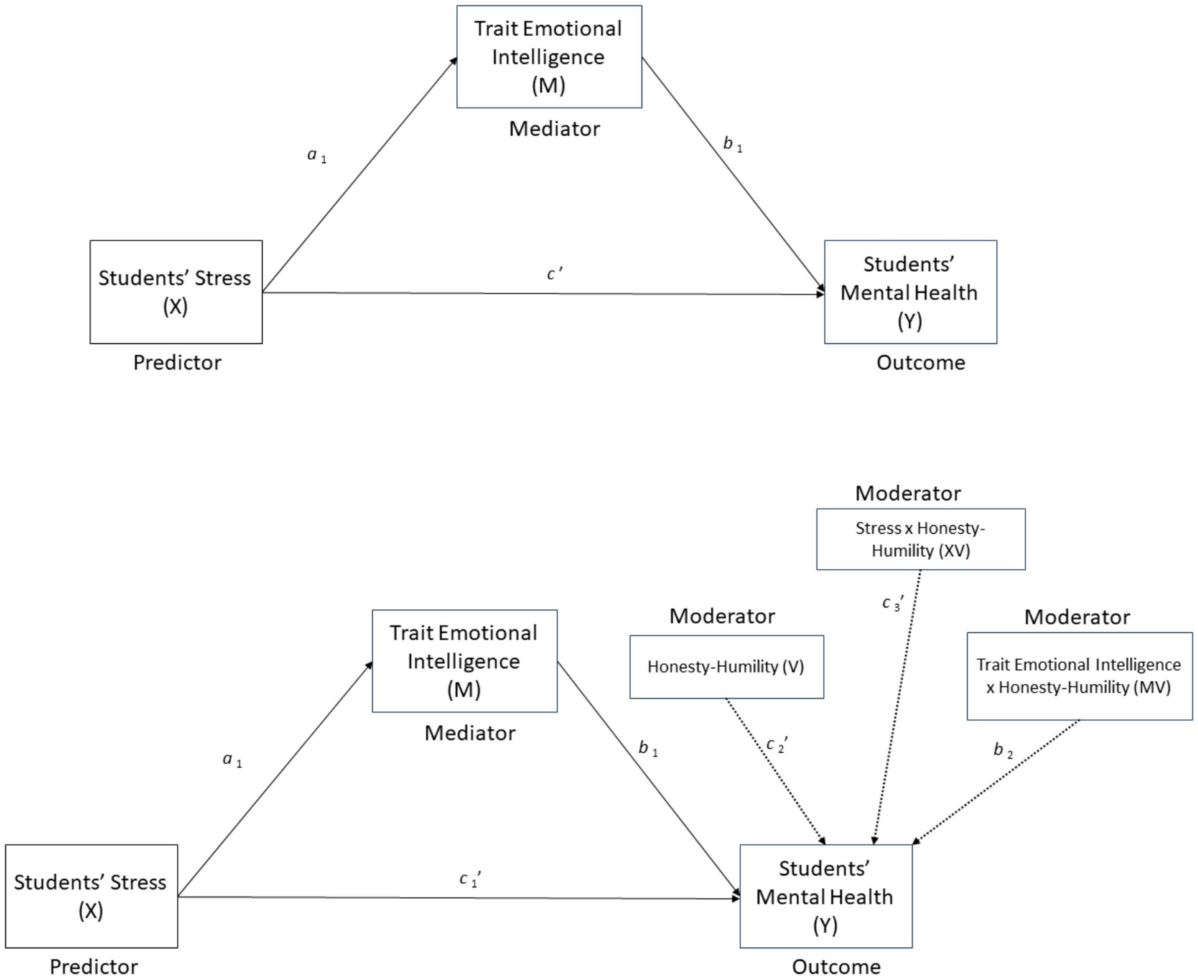
Statistical models were estimated using PROCESS. Standardized path coefficients and significance levels are reported along each path. *a*₁: Stress → Emotional Intelligence, *b*₁: Emotional Intelligence → Mental Health, *c*′: Direct effect (in Model 4), *c′₁, c*′₃: Direct + moderated paths (in Model 15), *b*₂: Interaction effect on the mediator path.

Descriptive and correlational analyses were conducted with SPSS version 29. In examining the relationships between students’ stress, trait emotional intelligence, and mental health, with an additional consideration of the moderating role of H-H, a sophisticated mediational model was employed. Utilizing the PROCESS Procedure for SPSS (Hayes, 2013), developed by Andrew F. Hayes, the analysis was conducted on a sample size of 746. The mediational model (Model 4) identified trait emotional intelligence as the mediator (M), with stress as the independent variable (X) and mental health as the dependent variable (Y). In a second step, Model 15 was used, considering H-H as a moderating variable (W), both on the direct (stress-mental health) and the indirect relationships (stress-trait emotional intelligence-mental health). The variables that define products were mean-centered before the analyses. The statistical Diagram is displayed in Figure 2. The top panel illustrates Model 4 (simple mediation), where trait emotional intelligence (M) serves as a mediator of the relationship between stress (X) and mental health (Y). The bottom panel illustrates Model 15 (moderated mediation), in which H-H (V) moderates both the direct (X → Y) and indirect (M → Y) pathways. Standardized path coefficients and significance levels are reported along each path.

## Results

### Descriptive statistics and Pearson’s correlations

As Table 1 showed, correlation analysis underscored a significant negative association between stress and both trait emotional intelligence and mental health, indicating that individuals with higher emotional intelligence tend to report lower stress levels and better mental health. Furthermore, a notable positive relationship emerged between trait emotional intelligence and mental health, indicating that emotional intelligence plays a key role in promoting psychological wellbeing. While the correlation between stress and H-H was weaker, it nonetheless suggested a pattern in which higher levels of H-H are associated with lower stress levels. Similarly, a positive correlation was observed between H-H and mental health, although it was less pronounced in magnitude. These findings illuminate the intricate web of relationships among personality traits, emotional intelligence, and psychological outcomes, highlighting the pivotal role of emotional intelligence in managing stress and promoting mental health.

**Table 1:** Descriptive statistics and Pearson’s correlation matrix (*N* = 746).

Variables	Mean	S.D.	Stress	Trait emotional intelligence	Honesty-humility	Mental health
Stress	2.59	0.756	*0.77*			
Trait emotional intelligence	5.00	0.731	−0.614^**^	*0.84*		
Honesty-humility	5.01	1.06	−0.192^**^	0.225^**^	*0.79*	
Mental Health	5.38	0.994	−0.619^**^	0.811^**^	0.131^**^	*0.80*

*p < 0.05; ***p < 0.001. Values in italics in the diagonal are Cronbach’s alphas.

### Hypothesis 1: students’ stress will predict students’ mental health

To test Hypothesis 1, which posited that students’ stress would significantly predict their mental health, a regression analysis was conducted. The results indicated that students’ stress was a significant predictor of their mental health, as shown in Table 2 (Model *R*^2^ = 0.376; *F*(1, 744) = 449.84; *p* < 0.001). This suggests that higher levels of stress are associated with poorer mental health outcomes among students.

**Table 2:** Regression analysis for the mediation model.

Outcome variable	Predictor	*B*	SE	*t*	*p*	95% CI	β
Trait emotional intelligence (M)	Students’ stress (X)	−0.59	0.03	−21.21	<0.001	[−0.65, −0.54]	−0.61
Students’ mental health (Y)	Students’ stress (X)	−0.26	0.03	−7.42	<0.001	[−0.32, −0.19]	−0.19
	Trait emotional intelligence (M)	0.94	0.04	26.41	<0.001	[0.87, 1.01]	0.69

### Hypothesis 2: trait emotional intelligence will mediate the relationship between students’ stress and mental health

Hypothesis 2 proposed that trait emotional intelligence would mediate the relationship between students’ stress and their mental health. A mediation analysis was performed using the PROCESS macro (Model 4) to examine this hypothesis.

The analysis revealed that students’ stress levels were a significant predictor of trait emotional intelligence, indicating that higher levels of stress were associated with lower levels of trait emotional intelligence. Additionally, trait emotional intelligence was a significant predictor of students’ mental health (R^2^ = 0.682; *F*(2, 743) = 796.72; *p* < 0.001). The direct effect of students’ stress on their mental health, when controlling for trait emotional intelligence, remained significant but was reduced, as shown in Table 3.

**Table 3:** Total, direct, and indirect effects of students’ stress on students’ mental health.

Effect type	Effect	SE	*t*	*p*	95% CI	*β*
Total effect (X → Y)	−0.81	0.04	−21.51	<0.001	[−0.89, −0.74]	−0.62
Direct effect (X → Y)	−0.26	0.03	−7.42	<0.001	[−0.32, −0.19]	−0.19
Indirect effect (X → M → Y)	−0.56	0.04			[−0.64, −0.49]	−0.42

*N = 746. X = Students’ stress, M = Trait Emotional Intelligence, Y = Students’ Mental Health. Bootstrap samples = 5000.*

The indirect effect of students’ stress on their mental health through trait emotional intelligence was also significant, with a 95% confidence interval that did not include zero (−0.4716, −0.3804), suggesting partial mediation. Overall, the results support both Hypothesis 1 and Hypothesis 2. Students’ stress negatively impacts their mental health, and this relationship is partially mediated by trait emotional intelligence. This suggests that while stress directly impacts mental health, a portion of its effect operates indirectly through its influence on trait emotional intelligence.

### Hypothesis 3: humility will moderate the direct relationship between students’ stress and mental health

To test Hypothesis 3, which proposed that humility would moderate the direct relationship between students’ stress and mental health, a moderated mediation analysis was conducted using the PROCESS macro (Model 15). The results indicated that the interaction between students’ stress and humility did not significantly predict mental health, as shown in Table 4.

**Table 4:** Moderated mediation analysis for the direct relationship between students’ stress and mental health.

Outcome variable	Predictor	*B*	SE	*t*	*p*	95% CI	*β*
Trait emotional intelligence (M)	Students’ stress (X)	−0.59	0.03	−21.21	<0.001	[−0.65, −0.54]	−0.61
Students’ mental health (Y)	Students’ stress (X)	−0.27	0.03	−7.74	<0.001	[−0.33, −0.20]	−0.27
	Trait emotional intelligence (M)	0.95	0.04	26.64	<0.001	[0.88, 1.02]	0.69
	Humility (W)	−0.06	0.02	−3.05	0.002	[−0.10, −0.02]	−0.06
	Interaction (Stress × Humility)	−0.04	0.03	−1.46	0.145	[−0.10, 0.02]	−0.04
	Interaction (Emotional Intelligence × Humility)	−0.08	0.03	−2.49	0.013	[−0.14, −0.02]	−0.08

**Figure 3:**
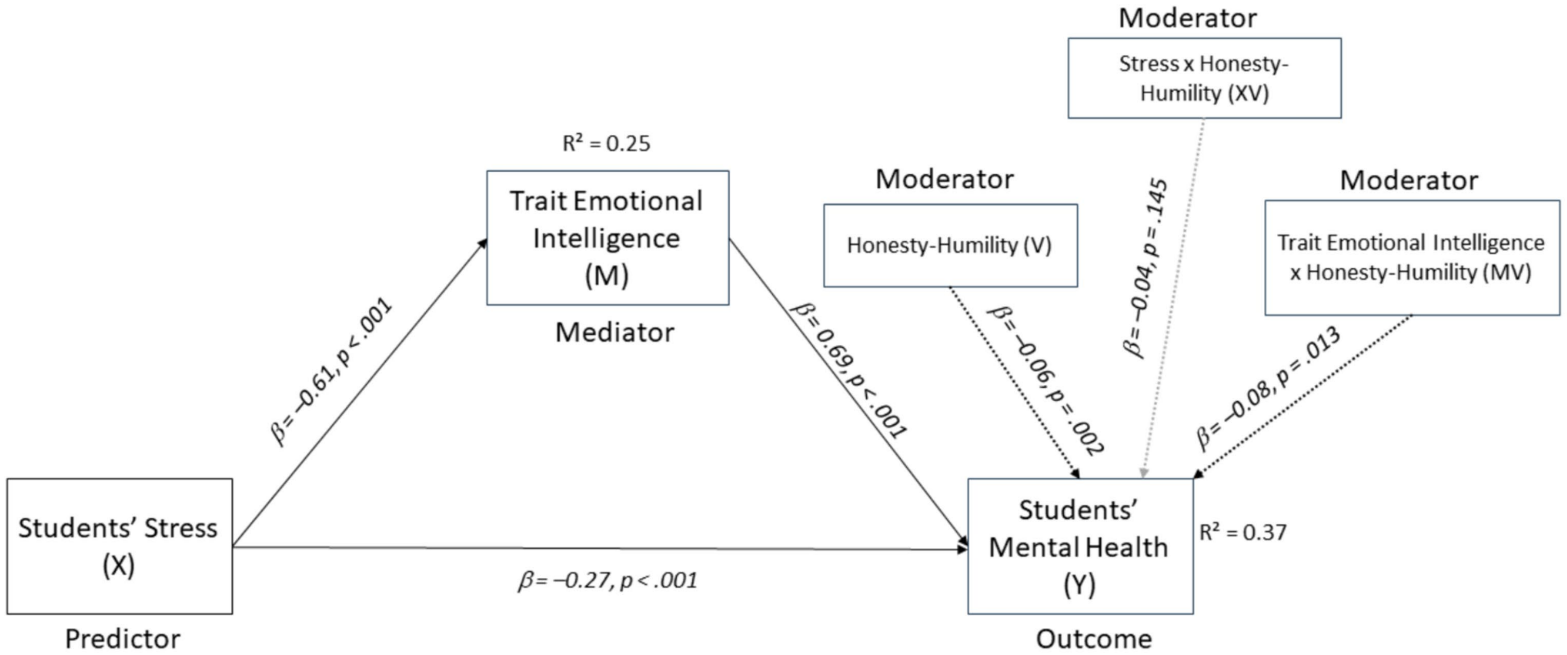
Final moderated mediation model (PROCESS Model 15). Dashed arrows indicate moderation effects. The gray arrow represents a non-significant interaction (*p* > 0.05). Standardized regression coefficients and *p*-values are shown. *R*^2^ values reflect the variance explained in each outcome variable.

The direct and indirect path coefficients are displayed in Figure 3.

**Figure 4:**
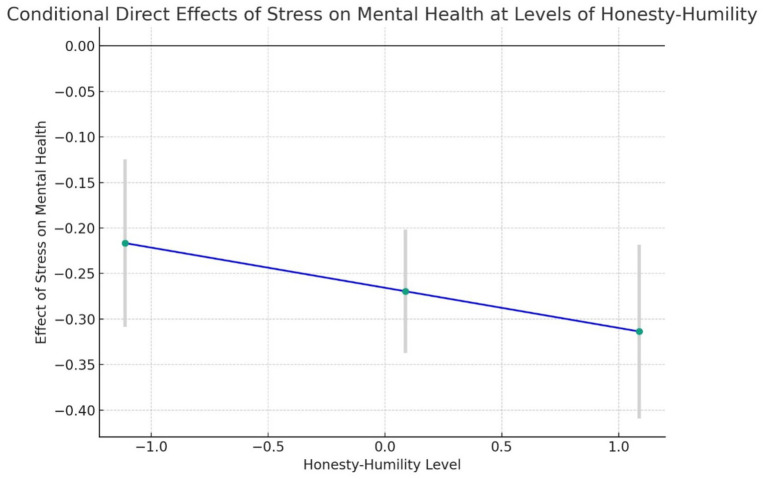
Line graph of conditional direct effects of students’ stress on mental health at different levels of honesty-humility. The graph illustrates how the effect of stress on mental health becomes more negative as the level of honesty-humility increases. Error bars represent the 95% confidence intervals, indicating the precision of these effect estimates.

However, the direct effect of students’ stress on their mental health was significant across all levels of humility, as shown in Table 5. These results suggest that while humility did not significantly moderate the direct relationship, the negative impact of stress on mental health was more pronounced at higher levels of humility, as shown in Figure 4.

**Table 5:** Conditional direct effects of students’ stress on mental health at different levels of humility.

Humility level	Effect	SE	*t*	*p*	95% CI	*β*
Low (−1.1121)	−0.22	0.05	−4.62	<0.001	[−0.31, −0.12]	−0.22
Average (0.0879)	−0.27	0.03	−7.81	<0.001	[−0.34, −0.20]	−0.27
High (1.0879)	−0.31	0.05	−6.46	<0.001	[−0.41, −0.22]	−0.31

### Hypothesis 4: humility will moderate the indirect relationship between students’ stress and mental health mediated by emotional intelligence

Hypothesis 4 posited that humility would moderate the indirect relationship between students’ stress and mental health through emotional intelligence. The Model displays adequate values, as indicated by Model R2 = 0.689, *F*(5, 740) = 327.36, *p* < 0.001. The results indicated a significant interaction between emotional intelligence and humility in predicting mental health, as shown in Table 6. This finding supports the hypothesis that humility moderates the indirect relationship.

**Table 6:** Index of Moderated Mediation.

Predictor	Index	BootSE	BootLLCI	BootULCI
Humility	0.0470	0.0200	0.0098	0.0878

BootSE = bootstrap standard error; BootLLCI = bootstrap lower-level confidence interval; BootULCI = bootstrap upper-level confidence interval. The indirect effects and index of moderated mediation indicate the role of humility in the indirect path from X to Y via the mediating variable (students’ stress on Mental Health through Trait Emotional Intelligence).

The index of moderated mediation was significant (see Table 6), indicating that the mediation of emotional intelligence is moderated by humility.

**Figure 5:**
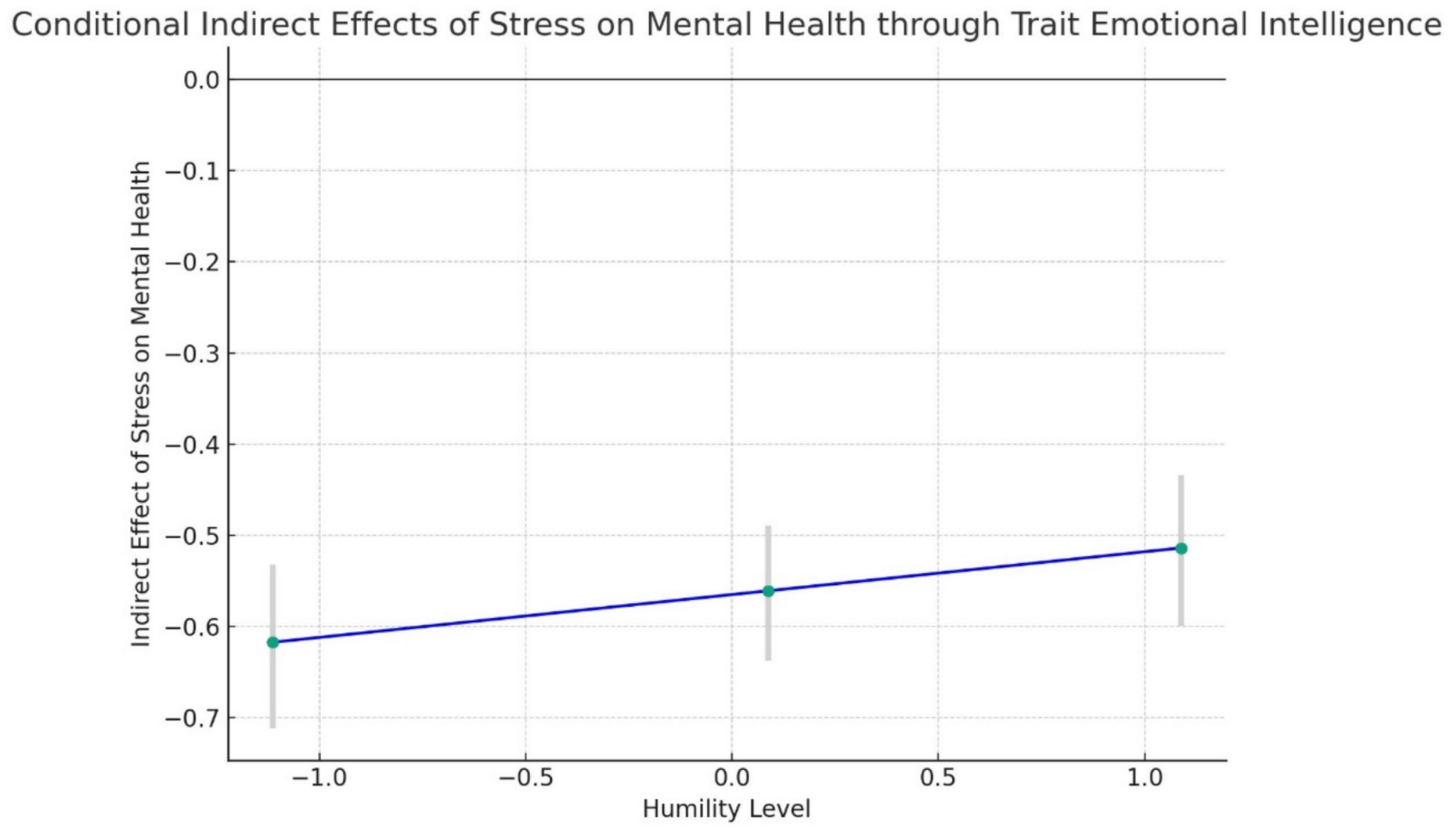
Line graph of the conditional indirect effects of students’ stress on mental health through trait emotional intelligence at different levels of humility. This graph illustrates that the indirect effect of stress on mental health becomes less negative as the level of humility increases. The error bars represent the 95% bootstrapped confidence intervals, indicating the reliability of these indirect effect estimates.

The conditional indirect effects of students’ stress on mental health through emotional intelligence were significant at all levels of humility. Specifically, at low levels of humility, the indirect effect was *β* = −0.6173; at average levels of humility, the indirect effect was *β* = −0.5609; and at high levels of humility, the indirect effect was *β* = −0.5139, as shown in Table 7. The results are depicted in Figure 5.

**Table 7:** Conditional indirect effects of students’ stress on mental health through trait emotional intelligence at different levels of humility.

Humility level	Indirect effect	BootSE	BootLLCI	BootULCI
Low (16th percentile)	−0.6173	0.0455	−0.7113	−0.5322
Average (50th percentile)	−0.5609	0.0378	−0.6372	−0.4888
High (84th percentile)	−0.5139	0.0421	−0.5991	−0.4341

*N = 746. Indirect effect values are derived from bootstrap estimates with 5,000 samples. BootLLCI = Bootstrapped lower limit confidence interval; BootULCI = Bootstrapped upper limit confidence interval.*

The moderation analyses provided insightful findings into the complex dynamics between stress, emotional intelligence, and mental health, as well as how the level of H-H influences these dynamics.

## Discussion

The present study aimed to examine the relationships among stress, mental health, and the HEXACO personality traits within the context of music education in China. Based on the present results, the mediational analysis provided substantial insights. Central to the findings was the direct negative influence of stress on mental health. The analyses revealed that trait emotional intelligence significantly mediated the relationship between stress and mental health across all examined levels of H-H (low, medium, and high). This pattern highlights a consistent indirect effect, indicating that increased stress levels have a detrimental impact on mental health, with this effect being mediated by the individual’s level of trait emotional intelligence, regardless of their H-H. This finding lends weight to the argument that emotional intelligence traits are pivotal in navigating the adverse effects of stress on mental health.

An individual with a relatively high level of emotional intelligence exhibits behaviors and characteristics that enable effective management of stress and its associated mental health challenges, particularly within the demanding context of music education. Such individuals are likely to possess a heightened ability to perceive and understand their own emotions, as well as those of others, which allows them to respond to stressors in a more adaptive and controlled manner. For example, they may utilize problem-focused coping strategies, such as breaking down tasks into manageable steps, or they might engage in emotion-focused coping, like reappraising stressful situations in a more positive light. Additionally, these individuals are better equipped to regulate their emotional responses, preventing stress from escalating into more severe mental health issues. This self-regulation not only helps them maintain emotional stability under pressure but also enables them to sustain their mental wellbeing over time. The mediation effect observed in this study highlights the importance of these emotional intelligence traits in mitigating the negative impact of stress, suggesting that interventions aimed at enhancing emotional intelligence could be particularly beneficial in supporting the mental health of students in high-stress educational environments, such as music education (Sacchetti and Salustri, 2023).

In line with these findings, and supporting the notion that emotional intelligence plays a crucial role in mediating stress and mental health, Wang and Zeb (2022) researched into the application of music education to improve college students’ mental health. Their research highlights the importance of musical emotions about individuals’ characteristics and cognitive processes, suggesting a beneficial impact of music education on mental wellbeing through enhanced emotional intelligence. In contrast, Liu et al. (2023) present findings that highlight the role of music training in enhancing personality traits, including extroversion, openness, agreeableness, and conscientiousness, which, in turn, impact psychological health. Their work suggests that while emotional intelligence is crucial, the broader spectrum of personality adjustments achieved through music training also significantly contributes to mental health outcomes.

In a related vein, Qi (2023) study on the impact of music therapy on university graduates facing psychological stress reinforces the importance of music as a therapeutic tool for regulating emotional and mental health. It aligns with the proposed model by providing empirical evidence on the effectiveness of music in managing psychological stress, thereby indirectly supporting the mediating role of emotional intelligence. On a different note, Leung and Cheung (2020) focus on the mediating processes between music engagement and wellbeing in Chinese adolescents. Their research highlights the mediation of emotional awareness and emotions between music training, listening, and adolescents’ wellbeing, suggesting a slightly different pathway through which music education influences mental health beyond the sole mediation of emotional intelligence.

Further complexity was unveiled through the moderated mediation analysis, which was marked by a significant index of moderated mediation. This outcome suggests that the mediational role of trait emotional intelligence in the stress-mental health nexus is not uniform but varies according to the level of H-H. Specifically, the conditional direct effects of stress on mental health also demonstrated significant variation across different levels of H-H. This variance highlights the complex interplay between stress, emotional intelligence, and personality traits in influencing mental health outcomes. These findings underscore the crucial importance of incorporating individual differences in emotional intelligence and personality traits into strategies designed to mitigate the adverse effects of stress on mental health, particularly within the demanding contexts faced by students in music education.

The moderation analyses within the study were designed to elucidate the roles of H-H in the interplay between student stress levels and mental health outcomes. Specifically, they are aimed to assess H-H’s influence on both the direct relationship between stress and mental health and the indirect relationship mediated by trait emotional intelligence.

Examining the conditional direct effects of stress on mental health across varying H-H levels revealed a nuanced pattern. As H-H increased, the adverse impact of stress on mental health became more pronounced. This trend indicates that individuals with higher levels of H-H may be more susceptible to the negative effects of stress on mental health, thereby affirming the hypothesis (H3) regarding H-H’s moderating role in this direct relationship.

Individuals with higher levels of H-H may be more susceptible to the negative effects of stress on mental health due to the inherent characteristics associated with this personality trait. Those high in H-H tend to exhibit behaviors such as sincerity, modesty, and a strong aversion to greed and dishonesty, which, while generally positive, may also make them more vulnerable in high-stress environments, such as music education. Their tendency toward fairness and modesty may lead to internalizing stress and avoiding assertive behaviors that could mitigate stress, such as seeking support or advocating for themselves. Furthermore, high H-H individuals might be less likely to engage in self-promoting or competitive behaviors that could help them manage the high demands of music education more effectively. This propensity for self-sacrifice and fairness, while ethically commendable, may limit their ability to cope with stress proactively, making them more susceptible to its negative impacts on mental health (Amponsah et al., 2024). The significant moderation effect observed in this study suggests that, while these individuals may possess a high moral character, their approach to stress management may lack the assertiveness and self-protective strategies necessary to buffer against the detrimental effects of stress, thereby exacerbating the impact of stress on their mental health outcomes.

These findings, while situated within the specific context of Chinese music education, point to broader psychological mechanisms that may generalize beyond this population. The interaction between H-H and emotional intelligence in shaping responses to stress underscores the importance of integrating ethical dispositions into models of emotional functioning. In particular, the diminished protective role of emotional intelligence at higher levels of H-H invites further inquiry into how self-regulatory and moral traits jointly influence psychological adaptation under pressure. Such an interaction has implications for understanding not only stress coping in education but also broader personality dynamics in socially demanding or ethically constrained environments.

Supporting the findings on the significant role of emotional intelligence in mediating stress and mental health outcomes, Kevereski et al. (2016) discovered that individuals with low emotional intelligence exhibit lower self-confidence and higher levels of depression and aggressiveness under stressful conditions. This underscores the crucial role of emotional intelligence in navigating the complexities of stress and mental health relationships, as observed in its mediating influence.

In contrast, Schutte et al. (2002) investigated whether emotional intelligence could moderate the relationship between stress and mental health outcomes. Their focus on emotional intelligence as a potential moderator echoes the importance of emotional intelligence in stress-related contexts. Still, it does not directly address the moderating impact of H-H, highlighting a gap in literature directly related to the study’s emphasis on H-H’s moderation. In a related vein, Jimenez Ballester et al. (2022) found happiness to be a relevant mediator between emotional intelligence and emotional symptoms in emerging adults, indicating the complexity of emotional processes and their significant impact on mental health. Although this does not directly affirm or refute the moderation analysis findings, it adds depth to the discourse on the mediating roles of emotional intelligence.

Turning the attention to the moderation of the indirect relationship by H-H, we focused on examining how H-H affects the mediation effect of trait emotional intelligence in the stress-mental health nexus. The index of moderated mediation provided clear evidence that H-H significantly moderates this indirect relationship. The conditional indirect effects of stress on mental health, as mediated through emotional intelligence, varied distinctly across different H-H levels. Notably, the mediation effect of emotional intelligence was found to be stronger at lower levels of H-H, with a gradual weakening observed as H-H increased. This pattern suggests that the protective role of emotional intelligence in buffering against the deleterious impact of stress on mental health is more potent among individuals with lower levels of H-H, lending support to the fourth hypothesis (H4).

The finding that the protective role of emotional intelligence (EI) in buffering against the deleterious impact of stress on mental health is more potent among individuals with lower levels of H-H offers intriguing insights into the interaction between these two traits. Individuals with lower levels of H-H may be more self-focused, assertive, and possibly more willing to engage in behaviors that prioritize their wellbeing, which could include utilizing their emotional intelligence to manage stress effectively. These individuals might be more inclined to use their emotional intelligence strategically, leveraging their ability to perceive, manage, and utilize emotions to navigate stressful situations in a way that minimizes the impact on their mental health. This strategic use of EI might be less constrained by the ethical and self-sacrificing tendencies that characterize those with higher H-H, allowing for more flexible and self-serving coping mechanisms.

Indeed, individuals with low H-H but high EI could be described as emotionally astute yet more opportunistic or pragmatic in their approach to stress management. They may possess the emotional skills to manipulate or control emotional situations to their advantage, using their EI to reduce stress while maintaining or enhancing their mental health. This combination of traits suggests a person who is not only aware of their own emotions and those of others but also willing to use this awareness in ways that are less bound by the ethical considerations typically associated with higher levels of H-H. This interpretation highlights the complex interplay between personality traits and emotional intelligence, suggesting that the effectiveness of EI as a protective factor against stress may be amplified in individuals who are less constrained by the moral and self-effacing characteristics associated with high H-H.

Regarding the specific role of H-H, a study by Lee and Seo (2018) on the effect of emotional intelligence on ego-resilience and self-efficacy touches on the broader implications of personality traits in emotional regulation and stress response. While not directly focused on H-H’s moderation effects, it underscores the relevance of examining personality dimensions in psychological research.

The findings from the moderation analyses illuminate the dynamics at play between stress, emotional intelligence, and mental health, further elucidated by the moderating influence of H-H. These results underscore the crucial importance of recognizing individual differences in personality traits, such as H-H, for a comprehensive understanding and effective intervention in the psychological effects of stress. The research suggests that mental health improvement strategies, particularly in stress-laden contexts, could achieve greater efficacy by tailoring approaches to cater to individuals’ specific levels of H-H and emotional intelligence.

In conclusion, the study makes a significant contribution to both the applied and theoretical domains by highlighting how individual differences in personality traits and emotional competencies jointly influence mental health outcomes under stress. While the primary aim was to inform mental health strategies for music students, the findings extend the current understanding of the complex roles played by H-H and emotional intelligence in psychological resilience. Future research should investigate these dynamics in other high-demand populations to assess the model’s generalizability and refine intervention strategies that are sensitive to personality-based differences in stress vulnerability and emotional regulation.

### Limitations and suggestions for future research

This study, which provides insightful findings on the relationships between stress, trait emotional intelligence, mental health, and the moderating role of H-H among Chinese music students, is not without limitations. Firstly, the sample is relatively homogeneous, consisting exclusively of music students from tertiary education institutions in China. This specificity may limit the generalizability of the findings to other populations, including non-music students, students in other disciplines, or individuals outside the academic context. Future research could benefit from examining these relationships in a broader demographic to enhance the generalizability of the findings.

Secondly, the study’s reliance on self-reported measures introduces the potential for response biases, such as social desirability bias or inaccuracies in self-assessment. Although instruments like the PSS-10, TEIQue-SF, HEXACO-60, and PMH-scale have demonstrated good reliability and validity, the inherent nature of self-report data can affect the objectivity of the results. Incorporating objective measures or third-party assessments could provide a more nuanced understanding of the constructs being examined.

The cross-sectional design of the study is another limitation, as it does not allow for the determination of causality between stress, trait emotional intelligence, mental health, and H-H. Longitudinal or experimental designs would be beneficial in establishing causal relationships and understanding the temporal dynamics of these variables.

In addition, although the study was conducted within the specific and meaningful context of music education, some of the emerging findings—particularly those related to the interaction between H-H and emotional intelligence—suggest broader theoretical implications. The unexpected pattern wherein the protective role of emotional intelligence appears stronger at lower levels of H-H raises questions about the functional and adaptive interplay between moral character and emotional regulation. Future studies should investigate whether this moderation pattern holds in other high-stress domains, such as healthcare, law enforcement, or competitive business environments, where similar personality dynamics may be at play.

Additionally, the exclusion of 43 individuals from the analysis due to incomplete survey responses may have introduced bias, potentially affecting the representativeness of the sample. This attrition could influence the study’s outcomes and interpretations, and future research may aim to minimize dropouts or account for their potential impact on the findings.

Furthermore, the study did not explore the specific subdimensions of H-H in depth, such as sincerity, fairness, greed avoidance, and modesty, about stress and mental health. Given the complexity and multidimensionality of the H-H construct, further research dissecting these subcomponents could reveal more detailed insights into how each aspect of H-H interacts with psychological outcomes.

Finally, while the study contributes to the understanding of the role of personality traits, such as H-H, in the context of stress and mental health among music students, it highlights the need for further research to explore these dynamics across diverse settings and populations. Expanding the scope of future studies to encompass diverse cultural backgrounds, educational disciplines, and age groups could provide a more comprehensive understanding of these important psychological relationships. Moreover, this study underscores the importance of integrating personality theory with emotional competence frameworks in future psychological research. While the applied relevance of supporting student mental health remains central, future investigations should also aim to refine theoretical models that account for the complex interactions between dispositional traits and stress regulation mechanisms. The findings here invite further exploration into the ethical, motivational, and regulatory dimensions of personality, which may influence not only mental health outcomes but also decision-making, interpersonal behavior, and coping strategies under pressure.

### Practical and policymakers’ implications

The exploration of stress, mental health, and emotional intelligence within the realm of music education presents significant practical implications for educators, students, and policymakers. Grounded in current research, these implications highlight the importance of integrating emotional intelligence training and mental health awareness into music education curricula to enhance both educational outcomes and student wellbeing.

For Practitioners and educators, the Emotional Skills Development approach should be applied. The role of music education in developing emotional skills is crucial. According to Campayo-Muñoz and Cabedo-Mas (2017), music education has a significant impact on emotional skill development, which in turn enhances wellbeing and cognitive performance. Educators should incorporate emotional intelligence training into music curriculums, leveraging music’s inherent emotional richness as a tool for teaching these skills effectively. Secondly, holistic and individualized instruction would be beneficial. Douwes et al. (2023) highlight the importance of adopting holistic and individualized instructional methods, especially for students with emotional and behavioral disorders. Music educators should tailor their teaching approaches to meet the diverse emotional and psychological needs of students, promoting an inclusive and supportive learning environment. Third, Music Therapy Integration could be proposed. The effectiveness of music therapy in alleviating psychological stress and promoting mental health, as evidenced by Qi (2023), suggests that music educators and therapists should collaborate to integrate music therapy practices into educational settings. This could provide students with additional resources to manage stress and enhance their emotional wellbeing. Finally, Self-Care for Educators is needed. The necessity of implementing self-care practices among music educators is underscored by Kuebel (2019). Educators should be equipped with knowledge and resources to manage their own stress and mental health, ensuring they can provide the best support for their students (Payne et al., 2020).

Regarding the policymakers, the main suggestions would be focused on the following points. First, policymakers should advocate for the inclusion of emotional intelligence and mental health components in music education curriculums. This would prepare students not only academically but also emotionally, equipping them with the skills necessary to navigate the challenges of their personal and professional lives. Second, allocating funding and resources for programs that integrate music education with emotional intelligence training and mental health awareness is essential. Investments in such programs can lead to improved educational outcomes and student wellbeing, contributing to the overall effectiveness of music education.

Third, policymakers should support professional development opportunities for music educators focused on emotional intelligence and mental health. Providing educators with the tools and knowledge to address these aspects within their teaching can enhance the educational experience for students. Fourth, continued research and evaluation of programs combining music education with emotional intelligence and mental health initiatives are crucial. Policymakers can support research efforts to better understand the impacts of these integrations and to inform future educational policies and practices.

In conclusion, the practical and policy implications of integrating stress management, mental health awareness, and emotional intelligence training into music education are manifold. By acknowledging and acting on these implications, educators and policymakers can significantly enhance the educational landscape for music students, fostering environments that support not only academic achievement but also emotional wellbeing and personal growth.

## Conclusion

In conclusion, this study makes a significant contribution to the understanding of the intricate relationships among personality traits—specifically, H-H—and emotional intelligence, stress, and mental health within the context of music education in China. The findings underscore the crucial mediating role of trait emotional intelligence in mitigating the adverse effects of stress on mental health among music students. Additionally, the moderation analysis reveals that the H-H dimension of personality has a significant influence on this mediated relationship, with higher levels of H-H potentially enhancing the protective effect of emotional intelligence against stress-related mental health challenges.

The practical implications of these findings are particularly relevant for music educators, students, and policymakers. Integrating emotional intelligence training into music education curricula could serve as a strategic approach to bolstering students’ resilience to stress, thereby improving their overall mental health and wellbeing. Moreover, recognizing the nuanced role of personality traits, such as honesty and humility, can guide the development of targeted interventions designed to support students in navigating the unique pressures inherent in music education.

However, this study is not without limitations. The cross-sectional design precludes definitive conclusions about causality, and the use of self-reported measures may introduce bias. Additionally, the sample was drawn from a specific cultural context, which may limit the generalizability of the findings to other settings. Future research should aim to replicate these findings in more diverse educational contexts and consider employing longitudinal designs to establish causal relationships better. Further investigation into the specific mechanisms through which H-H and emotional intelligence interact will also be valuable, potentially leading to more tailored and effective educational and psychological interventions.

Ultimately, this study paves the way for a deeper, more nuanced understanding of the psychological processes at play in music education. By fostering emotional intelligence and considering personality traits in educational practices, institutions can better support students’ mental health and contribute to their academic and personal success.

## Data Availability

The raw data supporting the conclusions of this article will be made available by the authors, without undue reservation.

## References

[ref1] Abbasi A. Z. Sundas A., & and Ting D. H. (2025). Validating the HEXACO Malay version as reflective-formative model: the application of hierarchical component model. Cogent Psychol., 12,:2466311. doi: 10.1080/23311908.2025.2466311

[ref2] Al-Dassean K. A. (2023). Psychometric properties of the Arabic version of the trait emotional intelligence questionnaire short form (TEIQue-SF). Cogent Psychol. 10:2171184. doi: 10.1080/23311908.2023.2171184

[ref3] Amponsah K. D. Adu-Gyamfi K. Awoniyi F. C. Commey-Mintah P. (2024). Navigating academic performance: unraveling the relationship between emotional intelligence, learning styles, and science and technology self-efficacy among preservice science teachers. Heliyon 10:e29474. doi: 10.1016/j.heliyon.2024.e29474, PMID: 38699017 PMC11064078

[ref4] Ashton M. C. Lee K. (2009). The HEXACO–60: a short measure of the major dimensions of personality. J. Pers. Assess. 91, 340–345. doi: 10.1080/00223890902935878, PMID: 20017063

[ref5] Bieda A. Hirschfeld G. Schönfeld P. Brailovskaia J. Zhang X. C. Margraf J. (2017). Universal happiness? Cross-cultural measurement invariance of scales assessing positive mental health. Psychol. Assess. 29, 408–421. doi: 10.1037/pas0000353, PMID: 27322203

[ref6] Campayo-Muñoz E. Á. Cabedo-Mas A. (2017). The role of emotional skills in music education. Br. J. Music Educ. 34, 243–258. doi: 10.1017/S0265051717000067

[ref7] Chen C. (2023). Mapping the terrain: a scoping review of empirical studies on the big five personality traits and quality of life in China. Front. Psychol. 14. doi: 10.3389/fpsyg.2023.1335657 PMC10811152 38282848

[ref8] Chen L. (2024). Delving into the role of self-efficacy in predicting motivation and engagement among music learners. Learn. Motiv. 86:101961. doi: 10.1016/j.lmot.2024.101961, PMID: 40194824

[ref9] Cohen S. Kamarck T. Mermelstein R. (1983). A global measure of perceived stress. J. Health Soc. Behav. 24, 385–396. doi: 10.2307/2136404, PMID: 6668417

[ref10] Cooper A. Petrides K. V. (2010). A psychometric analysis of the trait emotional intelligence questionnaire–short form (TEIQue–SF) using item response theory. J. Pers. Assess. 92, 449–457. doi: 10.1080/00223891.2010.497426, PMID: 20706931

[ref11] Demirbatir R. E. (2012). Undergraduate music student's depression, anxiety and stress levels: a study from Turkey. Procedia Soc. Behav. Sci. 46, 2995–2999. doi: 10.1016/j.sbspro.2012.05.603, PMID: 40194824

[ref12] Douwes R. Metselaar J. Pijnenborg G. H. M. Boonstra N. (2023). The well-being of students in higher education: the importance of a student perspective. Cogent Educ. 10:2190697. doi: 10.1080/2331186X.2023.2190697, PMID: 40101104

[ref13] Ewaiwi B. I. Attiyeh R. K. Niroukh E. A. Hijazi B. Y. Adawi S. O. Al-Qaissi H. S.. (2024). Emotional intelligence among medical students and residents in Palestine: a cross-sectional study. Migration Lett. 21, 1704–1715. doi: 10.22541/au.158809487.70957539

[ref14] Fiorilli C. Farina E. Buonomo I. Costa S. Romano L. Larcan R.. (2020). Trait emotional intelligence and school burnout: the mediating roles of resilience and academic anxiety in high school. Int. J. Environ. Res. Public Health 17:3058. doi: 10.3390/ijerph17093058 32354010 PMC7246930

[ref15] Fteiha M. Awwad N. (2020). Emotional intelligence and its relationship with stress-coping style. Health Psychol. Open 7:2055102920970416. doi: 10.1177/2055102920970416, PMID: 33224513 PMC7656878

[ref16] García L. F. Aluja A. Rossier J. Ostendorf F. Glicksohn J. Oumar B.. (2022). Exploring the stability of HEXACO-60 structure and the association of gender, age, and social position with personality traits across 18 countries. J. Pers. 90, 256–276. doi: 10.1111/jopy.12664, PMID: 34328208

[ref17] Hayes A. F. (2013). “Mediation, moderation, and conditional process analysis” in Introduction to mediation, moderation, and conditional process analysis: a regression-based approach, New York, NY: Guilford Publications. 1–20.

[ref18] Jääskeläinen T. (2023). “Music is my life”: examining the connections between music students’ workload experiences in higher education and meaningful engagement in music. Res. Stud. Music Educ. 45, 260–278. doi: 10.1080/14613808.2020.1841134

[ref19] Jimenez Ballester A. M. de la Barrera U. Schoeps K. Montoya-Castilla I. (2022). Emotional factors that mediate the relationship between emotional intelligence and psychological problems in emerging adults. Behav. Psychol. 30, 249–267. doi: 10.51668/bp.8322113n

[ref20] Kai C. (2012). “The study on mental health educational role of music education” in I*nformation and business intelligence. IBI 2011. Communications in Computer and Information Science*. eds. Qu X. Yang Y., vol. 267 (Berlin, Heidelberg: Springer).

[ref21] Kamza G. Bazarbekova R. (2023). The influence of music on the psycho-emotional state of students [СТУДЕНТТЕРДІҢ ПСИХОЭМОЦИОНАЛДЫҚ ЖАҒДАЙЫНА МУЗЫКАНЫҢ ӘСЕРІ]. J. Psychol. Sociol. 86, 27–35. doi: 10.26577/jpss.2023.v86.i3.0

[ref22] Kevereski L. Dimovska M. K. Ristevski D. (2016). Тhe influence of the emotional inteligence in protection of the mental health in conditions of a psychosocial stress. Int. J. Cognit. Res. Sci. Engin. Educ. 4, 17–21. doi: 10.5937/IJCRSEE1601017K

[ref23] Kuebel C. (2019). Health and wellness for in-service and future music teachers: developing a self-care plan. Music. Educ. J. 105, 52–58. doi: 10.1177/0027432119846950

[ref24] Lee Y.-J. Seo H.-J. (2018). The effect of emotional intelligence on Ego-resilience and self-efficacy. Korean J. Sport Stud. 57, 203–215. doi: 10.23949/kjpe.2018.11.57.6.15

[ref25] Leung M. C. Cheung R. Y. M. (2020). Music engagement and well-being in Chinese adolescents: emotional awareness, positive emotions, and negative emotions as mediating processes. Psychol. Music 48, 105–119. doi: 10.1177/0305735618786421

[ref26] Leung D. Y. Lam T.-h. Chan S. S. (2010). Three versions of perceived stress scale: validation in a sample of Chinese cardiac patients who smoke. BMC Public Health 10, 1–7. doi: 10.1186/1471-2458-10-513, PMID: 20735860 PMC2939644

[ref27] Li W. Wu J. Guo Z. Kou Y. (2023). Development of HEXACO personality traits and their relations with socioeconomic factors among Chinese adolescents: a three-wave longitudinal study. Eur. J. Personal. 37, 798–813. doi: 10.1177/08902070231174266

[ref28] Liu Y. Liu X. Zheng M. (2023). A correlation study of music training, adult attachment, and personality traits using a large-sample questionnaire. Front. Psychol. 14:1218848. doi: 10.3389/fpsyg.2023.1218848, PMID: 37691808 PMC10484518

[ref29] López-González M. A. Topa G. (2024). Exposome determinants of quality of life in adults over 50: personality traits, childhood conditions, and long-term unemployment in SHARELIFE retrospective panel. Psychol. Res. Behav. Manag. 17, 4207–4220. doi: 10.2147/PRBM.S472044, PMID: 39679320 PMC11645904

[ref30] Lu W. Bian Q. Wang W. Wu X. Wang Z. Zhao M. (2017). Chinese version of the perceived stress Scale-10: a psychometric study among Chinese university students. PLoS One 12:e0189543. doi: 10.1371/journal.pone.0189543, PMID: 29252989 PMC5734731

[ref31] Lukat J. Margraf J. Lutz R. van der Veld W. Becker E. (2016). Psychometric properties of the positive mental health scale (PMH scale). BMC Psychol. 4, 1–14. doi: 10.1186/s40359-016-0111-x 26865173 PMC4748628

[ref32] Mellor S. Elliott R. (2025). A construct validity study for the humility at work scale: item-content validity and convergent-discriminant validity. Merits 5:5. doi: 10.3390/merits5010005

[ref33] Molero-Jurado M. Perez-Fuentes M. Martinez Martos A. Barragán Martin A. Simon Marquez M. Gazquez Linares J. J. (2021). Emotional intelligence as a mediator in the relationship between academic performance and burnout in high school students. PLoS One 16:e0253552. doi: 10.1371/journal.pone.0253552, PMID: 34166434 PMC8224948

[ref34] Nwokenna E. N. Sewagegn A. A. Falade T. A. (2022). Effects of educational music training on music performance anxiety and stress response among first-year undergraduate music education students. Medicine 101:e32112. doi: 10.1097/MD.0000000000032112, PMID: 36482613 PMC9726349

[ref35] Payne P. D. Lewis W. McCaskill F. (2020). Looking within: an investigation of music education majors and mental health. J. Music. Teach. Educ. 29, 50–61. doi: 10.1177/1057083720927748, PMID: 40182056

[ref36] Petrides K. V. Furnham A. (2000). On the dimensional structure of emotional intelligence. Personal. Individ. Differ. 29, 313–320. doi: 10.1016/S0191-8869(99)00195-6

[ref37] Pilch I. (2023). Comparison of the big five and the HEXACO models of personality in the prediction of emotional wellbeing: an experience sampling study. Trends Psychol., 1–17. doi: 10.1007/s43076-023-00311-w, PMID: 40192768

[ref38] Qi S. (2023). An exploration of music therapy on relieving psychological stress of graduates. Lecture Educ. Psychol. Public Media 27, 216–220. doi: 10.54254/2753-7048/27/20231191, PMID: 40188479

[ref39] Sacchetti S. Salustri A. (2023). Teaching and playing? A survey on the well-being and motivations of young musicians. Merits 3, 521–537. doi: 10.3390/merits3030031

[ref40] Schutte N. S. Malouff J. M. Simunek M. McKenley J. Hollander S. (2002). Characteristic emotional intelligence and emotional well-being. Cognit. Emot. 16, 769–785. doi: 10.1080/02699930143000482

[ref41] Sun J. (2022). Exploring the impact of music education on the psychological and academic outcomes of students: mediating role of self-efficacy and self-esteem. Front. Psychol. 13:841204. doi: 10.3389/fpsyg.2022.841204 35211068 PMC8863131

[ref42] Sun G. Lyu B. (2022). Relationship between emotional intelligence and self-efficacy among college students: the mediating role of coping styles. Discov. Psychol. 2:42. doi: 10.1007/s44202-022-00055-1

[ref43] Travis J. Neale C. A. Wilgus S. J. (2024). When dark personality gets darker: the intersection of injustice, moral disengagement, and unethical decision making. Merits 4, 414–430. doi: 10.3390/merits4040029

[ref44] Wang H. Jia R. Zhang M. Fan W. (2024). The influence of stress on mental health among Chinese college students: the moderating role of psychological Suzhi. Heliyon 10:e26699. doi: 10.1016/j.heliyon.2024.e26699, PMID: 38444499 PMC10912246

[ref45] Wang F. Zeb S. (2022). Impact of music education on mental health of higher education students: moderating role of emotional intelligence. Front. Psychol. 13:938090. doi: 10.3389/fpsyg.2022.938090, PMID: 35783702 PMC9240095

[ref46] Widodo W. Gustari I. Chandrawaty C. (2022). Adversity quotient promotes teachers’ professional competence more strongly than emotional intelligence: evidence from Indonesia. J. Intelligence 10:44. doi: 10.3390/jintelligence10030044, PMID: 35893275 PMC9326609

[ref47] Wristen B. G. (2013). Depression and anxiety in university music students. *Update: applications of research in music*. Education 31, 20–27. doi: 10.1177/8755123312473613, PMID: 40182056

[ref48] Wu Y. Sang Z.-q. Zhang X.-C. Margraf J. (2020). The relationship between resilience and mental health in Chinese college students: a longitudinal cross-lagged analysis. Front. Psychol. 11:450850. doi: 10.3389/fpsyg.2020.00108 PMC7012791 32116918

[ref49] Zarei F. Akbarzadeh I. Khosravi A. (2019). The relationship between emotional intelligence and stress, anxiety, and depression among Iranian students. Int. J. Health Stud. 5. doi: 10.22100/ijhs.v5i3.668

[ref50] Zhang Y. Hu J. (2024). Fatalism and depressive symptoms among Chinese college students: mediation models of locus of control and positive coping. Heliyon 10:e27617. doi: 10.1016/j.heliyon.2024.e27617 38509900 PMC10950598

[ref51] Zhang Y. Luo X. Che X. Duan W. (2016). Protective effect of self-compassion to emotional response among students with chronic academic stress. Front. Psychol. 7:218738. doi: 10.3389/fpsyg.2016.01802, PMID: 27920736 PMC5118418

